# Changes in the Intracellular Composition of Macro and Microminerals After Cryopreservation of the Rabbit Stem/Progenitor Cells

**DOI:** 10.3390/jdb13010006

**Published:** 2025-02-21

**Authors:** Jaromír Vašíček, Andrej Baláži, Mária Tirpáková, Marián Tomka, Peter Chrenek

**Affiliations:** 1 National Agricultural and Food Center, Research Institute for Animal Production Nitra, Hlohovecká 2, 951 41 Lužianky, Slovakia; andrej.balazi@nppc.sk (A.B.); peter.chrenek@uniag.sk (P.C.); 2 Faculty of Biotechnology and Food Science, Slovak University of Agriculture in Nitra, Tr. A. Hlinku 2, 949 76 Nitra, Slovakia; marian.tomka@uniag.sk; 3 AgroBioTech Research Center, Slovak University of Agriculture in Nitra, Tr. A. Hlinku 2, 949 76 Nitra, Slovakia; maria.tirpakova93@gmail.com

**Keywords:** rabbit, stem cells, cryopreservation, minerals, ICP-OES

## Abstract

Cryopreservation is a widely used method for the long-term preservation of reproductive or somatic cells. It is known that this storage method may negatively affect cell viability, proliferation, differentiation, etc. However, there is a lack of information about whether cryostorage can alter the content of intracellular minerals. Therefore, we focused this study on the analysis of the mineral composition of living cells before and after long-term cold storage. Briefly, three different primary cell lines were established from rabbits as follows: endothelial progenitor cells from peripheral blood (EPCs), endothelial progenitor cells from bone marrow (BEPCs), and mesenchymal stem cells from adipose tissue (AT-MSCs), which were cultured until passage 3 prior to cryopreservation in liquid nitrogen. Samples from freshly cultured and frozen–thawed cells were mineralized and analyzed using inductively coupled plasma-optical emission spectroscopy (ICP-OES) for the content of minerals (macro: Ca, Na, K, and Mg, and micro: Zn, Fe, Cu, Al, Co, Mn, Sr, and Ni). After cryopreservation, we found significantly decreased content of K in frozen–thawed EPCs (*p* < 0.01) and BEPCs (*p* < 0.0001) and Ca in AT-MSCs (*p* < 0.05), while Na was increased in frozen–thawed BEPCs (*p* < 0.05). Concentrations of Fe and Al were reduced significantly in frozen–thawed EPCs (both *p* < 0.0001) and AT-MSCs (*p* < 0.001 and *p* < 0.0001, respectively). On the contrary, Fe and Al were elevated in frozen–thawed BEPCs (*p* < 0.0001 and *p* < 0.01, respectively) together with Ni (*p* < 0.0001). In addition, decreased Zn (*p* < 0.05) was observed in cryopreserved AT-MSCs. In conclusion, the ICP-OES technique might be used to analyze the basic elemental composition of animal cells in fresh or frozen–thawed conditions. Nevertheless, additional studies are needed to reveal the possible impact of cryopreservation on cell fate by changing the content of intracellular minerals.

## 1. Introduction

Living organisms are constituted by different organic and inorganic chemicals, as well as molecules, which finally form the cells in tissues. Animal cells, excluding some specialized cells (such as those of feathers, hair, bones, hooves, horns, cartilage, or dermis), consist, in general, of water (80%) and solids (20%), which are mainly made up of organic compounds (99%; carbon, hydrogen, oxygen, and nitrogen) and very small amounts of inorganic compounds (<1%; water, acid, bases, salts, minerals, etc.). In fact, minerals presented in animal bodies are comprised of seven major macrominerals (calcium, sodium, potassium, chlorine, phosphorus, magnesium, and sulfur) and more than twenty microminerals or trace elements. Thirteen microminerals are essential or beneficial, such as copper, cobalt, iron, iodine, zinc, fluorine, molybdenum, manganese, selenium, silicon, strontium, chromium, and boron, and eight of them are probably essential as aluminum, arsenic, bromine, lithium, lead, nickel, tin, and vanadium [1,2].

Each of the macro or microminerals plays a very significant role in the living body as a part of important metabolic functions. In the case of microminerals, calcium (Ca) is involved in the mineralization of bones, cell signaling, blood clotting, contraction of muscles, or transmission of nerve impulses. Phosphorus (P) also participates in bone mineralization and the regulation of key regulatory enzymes. Furthermore, P is a component of nucleic acids (RNA and DNA), high-energy compounds (ATP and others), and phospholipids. Magnesium (Mg) is known to be a cofactor for over 300 enzymes and is also another component of bones. Sodium (Na) participates in the regulation of osmotic pressure and acid–base balance as well as in the active transport of nutrients, conduction of nerves, and contraction of muscles. Chlorine (Cl) also regulates osmotic pressure and acid-base balance, controls the balance of water, and forms HCl in gastric juice. Potassium (K) similarly takes part in the regulation of acid-base balance and osmotic pressure and controls water balance, muscle contraction, and transmission of nerve impulses. Sulfur (S) is a component of sulfur amino acids, thiamin, biotin, and sulfated mucopolysaccharides. In addition, S is involved in detoxification processes. Microminerals are important components of enzymes, vitamins, or hormones. Cobalt (Co) is a component of cobalamin, which is a functional part of vitamin B_12_. Copper (Cu) is incorporated into various enzymes such as lysyl oxidase, ceruloplasmin, tyrosinase, cytochrome oxidase, superoxide dismutase, etc. Iodine (I) is a component of thyroid hormones (thyroxine and triiodothyronine). Iron (Fe) takes part in the transport and storage of oxygen (hemoglobin and myoglobin), in the transport of electrons, and is a part of numerous enzymes such as catalase, tryptophan, 5-monoxygenase, phenylalanine 4-monoxygenase aconitase, etc. Manganese (Mn) is an activator of enzymes (e.g., glycosyl transferases) as well as an enzyme component (pyruvate carboxylase, arginase, and mitochondrial superoxide dismutase). Molybdenum (Mo) is present in xanthine, sulfite, and aldehyde oxidase. Selenium (Se) is incorporated into important antioxidant enzymes (glutathione peroxidase, thioredoxins, etc.). Zinc (Zn) is involved in the expression of genes and the stability of membranes. Moreover, it is part of about 100 enzymes such as RNA and DNA polymerase, alcohol and pyruvate dehydrogenase, carbonic anhydrase, carboxypeptidase A and B, etc. [2,3,4,5]. Aluminum (Al) is the third most abundant mineral in the lithosphere and can be partially found in all living organisms. Despite this fact, there is no evidence about it participating in any bodily biological or biochemical processes [6,7]. Strontium (Sr) is known to be involved in bone formation and mineralization [8]. Nickel (Ni) is utilized by bacteria, lower eukaryotes, and plants as a necessary component in essential metallo-enzymes. However, such enzymes or cofactors were not found in higher animals [9]. On the other hand, many animal studies showed nickel to be beneficial or even essential for the reproductive system, composition, and strength of bones, sensory function, or energy metabolism [10].

Inductively coupled plasma-optical emission spectroscopy (ICP-OES) has become a widely preferred technique to detect and quantify chemical elements mainly due to its ability to measure numerous elements simultaneously [11,12]. In this method, excited atoms and ions are generated using inductively coupled plasma, which subsequently emit electromagnetic radiation typical for each element [13]. Nowadays, the ICP-OES method is extensively used in both the industrial and academic sectors and may be applied for elemental analysis in microplastics, food, dietary supplements, and even biological systems [12]. For example, the amounts of micro (Mn, Co, Cu, Zn, As, Se, Cd, Pb, and U) and macroelements (Ca, Na, K, and Mg) have been assessed in human adrenal tissues and peripheral blood samples using ICP-OES analysis [14]. Moreover, several studies have been published studying the cellular uptake of different elements, such as ruthenium, platinum, gallium, silver, or iron, etc., by various cell lines [15,16,17,18,19,20] or cell efflux of, e.g., potassium during inflammasome activation [21].

Cryopreservation is a standard method for long-term storage of any cell type, including reproductive cells such as spermatozoa and oocytes, as well as somatic cells comprising mature cells or stem and progenitor cells of different tissues. Generally, it has been observed that the cryopreservation process itself, due to the considerable temperature changes and chemical composition of used cryoprotectants, may affect different cell attributes such as survival rate, overall viability, apoptosis, proliferation potential, or capacity for differentiation [22]. Interestingly, an influx of calcium into sperm cytoplasm was observed concurrently with the decrease in temperature during the cryopreservation process [23,24]. Another study indicated a possible increase in calcium levels in cryopreserved porcine cardiovascular tissue [25]. On the other hand, levels of selected minerals (Al, B, Ba, Ca, Cu, Fe, K, Mg, Mn, Na, Ni, P, S, Sr, and Zn) were not affected by cryopreservation of the pineapple plant tissue [26]. However, as far as we know, the possible effect of cryopreservation on the elemental composition of animal somatic or stem cells has not yet been thoroughly studied.

The aim of the presented study was to observe if cryopreservation may alter the concentration of elements in long-term cryo-stored rabbit primary cells.

## 2. Materials and Methods

### 2.1. Animals

Clinically healthy female rabbits of the New Zealand White line (*n* = 6) at the age of 3–6 months old were used in this study. The rabbits were reared at NPPC—Research Institute for Animal Production Nitra (NPPC-RIAP Nitra) under conditions described in a previous study [27]. All experiments were performed with the approval of the Ministry of Agriculture and Rural Development of the Slovak Republic no. SK U 18016 in accordance with the ethical guidelines presented in the Slovak Animal Protection Regulation (RD 377/12), which conforms to the Code of Ethics of the EU Directive 2010/63/EU for animal experiments.

### 2.2. Collection of Biological Material and Isolation of Rabbit Primary Cells

The collection of peripheral blood, bone marrow, and subcutaneous fat from rabbits was performed as described previously [28,29]. Briefly, mononuclear cells from blood and bone marrow were isolated using density-gradient centrifugation in a Biocoll separating solution (Biochrom, Berlin, Germany). Cells were resuspended in a basal medium specific for endothelial cells (EBM-2) containing growth factors (EGM-2 medium; Lonza, Walkersville, MD, USA), fetal bovine serum (FBS; Sigma Aldrich, Gillingham, UK), and a penicillin/streptomycin solution (Thermo Fisher Scientific, Waltham, MA, USA), and seeded into T75 culture flasks. Cells were cultured at 37 °C in a 5% CO_2_ humidified atmosphere until passage 3 to obtain a pure population of rabbit endothelial cells derived from peripheral blood (EPCs) or bone marrow (BEPCs).

To obtain a single-cell suspension, adipose tissue samples from subcutaneous fat were dissociated using enzyme collagenase type I (Sigma Aldrich, Gillingham, UK). After filtration, cells were seeded to T75 flasks in α-MEM culture medium (Gibco^TM^, Thermo Fisher Scientific) containing FBS and antibiotics, as mentioned above, and cultured until passage 3 to obtain adipose-tissue derived mesenchymal stem cells (AT-MSCs).

At least three cultures from each of the rabbit primary cell lines (EPCs, BEPCs, and AT-MSCs) were established and subsequently used for further ICP-OES procedures.

### 2.3. Preparation and Storage of Cell Samples for ICP-OES Analysis

Cells at passage 3 from each culture (EPCs, BEPCs, and AT-MSCs) with confluency at 80–90% were harvested using 0.05% Trypsin- EDTA (Thermo Fisher Scientific) and counted by EVE™ Automatic cell counter (NanoEntek, Seoul, Republic of Korea). One-half of each sample was processed as a “fresh sample” by washing twice in a 150 mM Tris-HCl solution and counted again. Afterward, fresh cell samples were centrifuged at 500× *g* for 5 min at 20 °C. After aspirating the supernatant, each cell pellet was frozen at −80 °C for further ICP-OES analysis. The second half of each sample was frozen in a corresponding culture medium containing 10% DMSO and stored in liquid nitrogen for at least three months. After that, frozen samples were thawed, washed twice in a 150 mM Tris-HCl solution, and counted. The pellets from “frozen–thawed samples” were centrifuged and frozen without supernatant at −80 °C for further ICP-OES analysis.

### 2.4. Sample Mineralization and ICP-OES Analysis

Prior to ICP-OES analysis, samples (pellets of fresh and frozen–thawed cells) stored at −80 °C were defrosted at room temperature and mineralized using the high-performance microwave digestion system Ethos UP (Milestone Srl, Sorisole, BG, Italy) using a solution of 8 mL of HNO_3_ (TraceSELECT^®^, Honeywell Fluka, Morris Plains, NJ, USA) and 2 mL of H_2_O_2_ (30%, for trace analysis, Merck Suprapur^®^; Merck KGaA, Darmstadt, Germany). Blank samples and experimental samples were digested according to the preloaded method “animal tissue” developed by the manufacturer to ensure the best result. At first, samples were heated for 15 min up to 200 °C, keeping this temperature for 15 min, and subsequently, samples underwent active cooling for an additional 15 min. The final digests were cooled to 50 °C and filtered through the Sartorius filter discs (grade 390) (Sartorius AG, Goettingen, Germany) into the volumetric flask. At last, samples were filled up with ultrapure water to a volume of 50 mL.

The concentration of selected elements (intracellular macrominerals: Ca, Na, K, and Mg, and microminerals: Zn, Fe, Cu, Al, Co, Mn, Sr, and Ni) was analyzed using inductively coupled plasma optical emission spectrophotometer ICP Thermo ICAP 7000 Dual (Thermo Fisher Scientific). The detection limits (µg/L) of measured minerals were as follows: Ca 0.29, Na 185.00, K 621.00, Mg 0.40, Zn 0.50, Fe 1.20, Cu 3.30, Al 7.70, Co 1.80, Mn 0.30, Sr 2.00, and Ni 1.70. The operation conditions of the instrument are listed in Table 1.

Multielement standard solution V for ICP (Sigma Aldrich) was used for calibration, and the accuracy of element determination was assessed using certified reference material (ERM-CD281, Sigma Aldrich). All samples were measured in three replicates. The obtained results were recalculated to the concentration of the analyzed element in ng per 10^6^ cells according to Equation (1):

(1) $$\frac{weight\;of\;mineralized\;compound\times element\;concentration\;(\mu g/L)}{cell\;concentration\;(10^{6})}=x\;ng/10^{6}\;cells$$

### 2.5. Statistical Analyses

Obtained data were statistically assessed by GraphPad Prism version 9.5.1 for Windows (GraphPad Software, San Diego, CA, USA) with a two-way ANOVA followed by Fisher’s LSD test. Results are expressed as the mean ± SD. *p*-values at *p* < 0.05 were considered statistically significant.

## 3. Results

**Figure 1:**
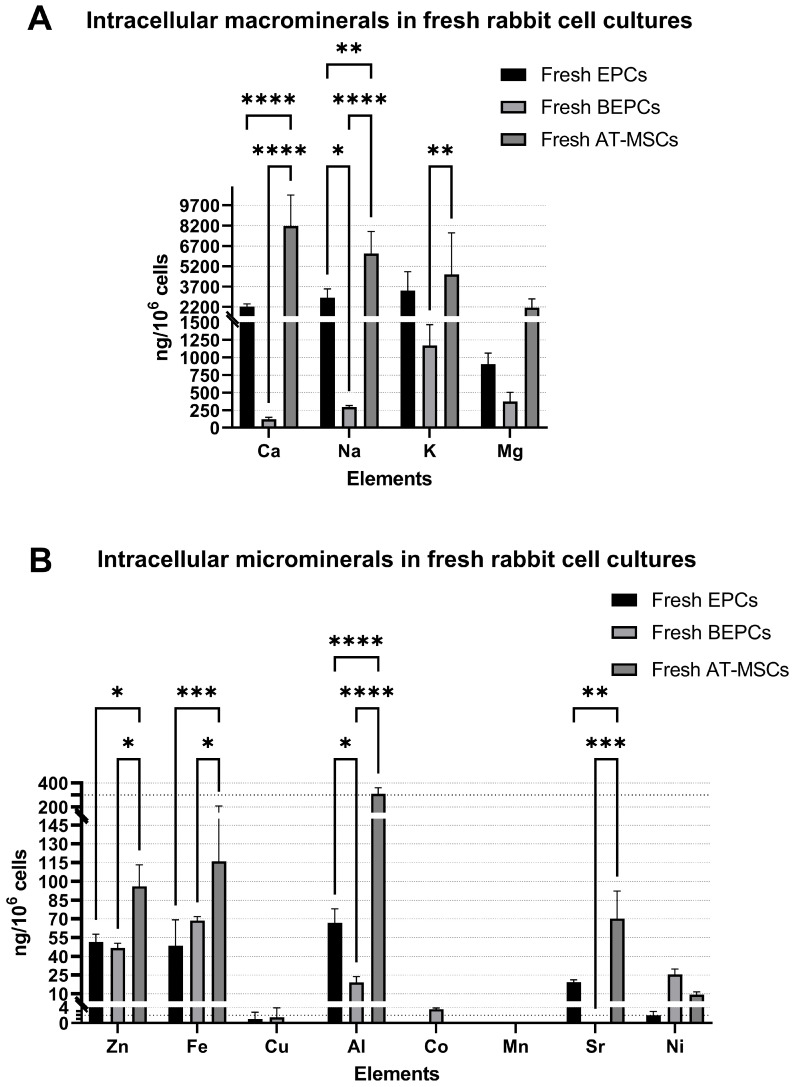
Comparison of the macro and micromineral concentrations presented in the three rabbit fresh cell cultures (**A**,**B**). EPCs—rabbit endothelial progenitor cells derived from peripheral blood, BEPCs—rabbit endothelial progenitor cells derived from bone marrow, AT-MSCs—rabbit adipose-tissue derived mesenchymal stem cells. The data from three independent cell cultures for each cell line are expressed as the means ± SD; *—the difference is statistically significant at *p* < 0.05; **—the difference is statistically significant at *p* < 0.01; ***—the difference is statistically significant at *p* < 0.001; ****—the difference is statistically significant at *p* < 0.0001. Measurements for Cu (AT-MSCs), Co (EPCs and BEPCs), Mn (all cell lines), and Sr (BEPCs) were under the detection limits.

In this study, the elemental composition of three different rabbit primary cell cultures was assessed in fresh and cryopreserved samples. In fresh cell cultures, statistically significant differences were found for the concentration of Ca, Na, and K (except for Mg) among rabbit EPCs, BEPCs, and AT-MSCs. The highest concentrations of those three macrominerals (>4000 ng/10^6^ cells) were observed in fresh AT-MSCs (*p* < 0.05). Interestingly, fresh EPCs contained much more Ca and Na than fresh BEPCs (*p* < 0.05; Figure 1A). In the case of microminerals, fresh AT-MSCs again exhibited the highest concentration of several elements, such as Zn, Fe, Al, and Sr (*p* < 0.05), compared to other cell cultures. A trace amount (<30 ng/10^6^ cells) of other analyzed microminerals (Cu, Co, Mn, and Ni) did not significantly differ among the rabbit fresh cell cultures (Figure 1B).

**Figure 2:**
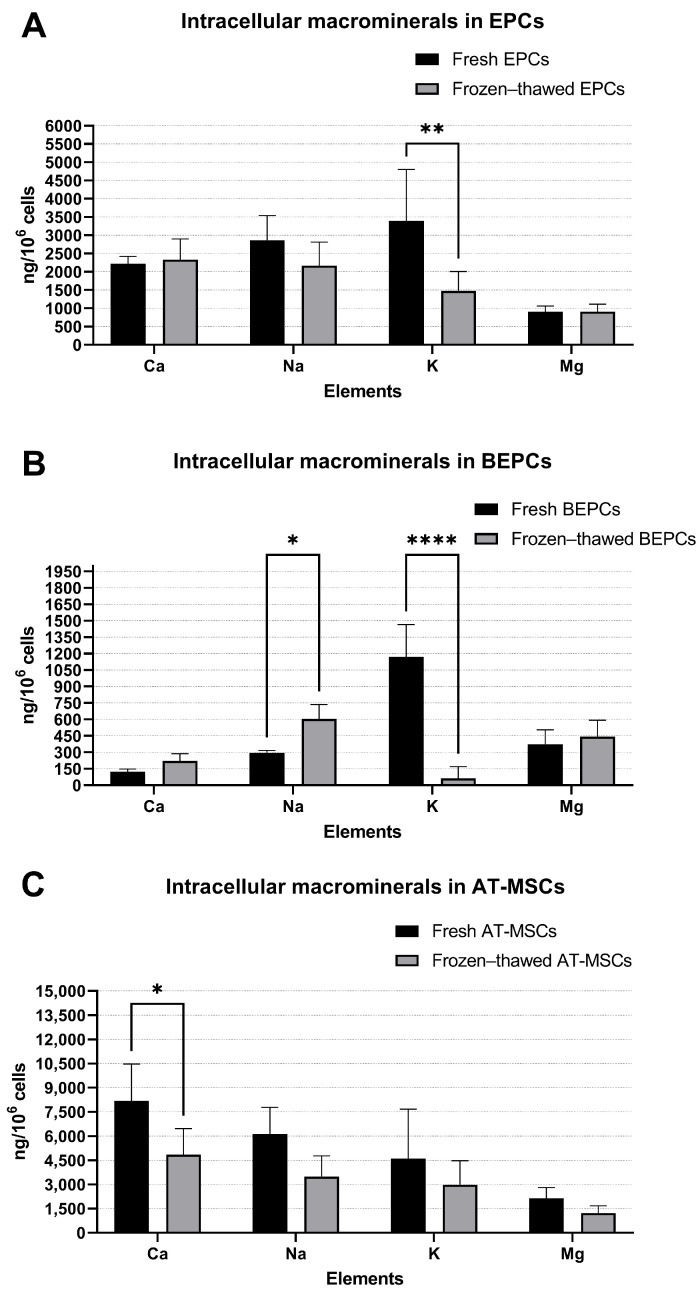
Effect of cryopreservation on the composition of macrominerals in the rabbit primary cell lines (**A**–**C**). EPCs—rabbit endothelial progenitor cells derived from peripheral blood, BEPCs—rabbit endothelial progenitor cells derived from bone marrow, AT-MSCs—rabbit adipose-tissue derived mesenchymal stem cells. The data from three independent cell cultures for each cell line are expressed as the means ± SD; *—the difference is statistically significant at *p* < 0.05; **—the difference is statistically significant at *p* < 0.01; ****—the difference is statistically significant at *p* < 0.0001.

In the case of cryopreserved samples, we noticed a decrease in the level of K (*p* < 0.01) and an insignificant decrease in Na level for EPC samples compared to fresh samples (Figure 2A), while the concentration of other macrominerals (Ca and Mg) did not change. In BEPCs, the cryopreservation process similarly decreased the concentration of K (*p* < 0.0001), whereas the level of Na increased (*p* < 0.05). Other analyzed macrominerals (Ca and Mg), again, did not change (Figure 2B). On the other hand, the level of Ca decreased (*p* < 0.05) in AT-MSCs after cryopreservation compared to fresh samples (Figure 2C). The concentration of other macrominerals (Na, K, and Mg) was not affected by the cryopreservation of AT-MSCs, although they decreased insignificantly.

**Figure 3:**
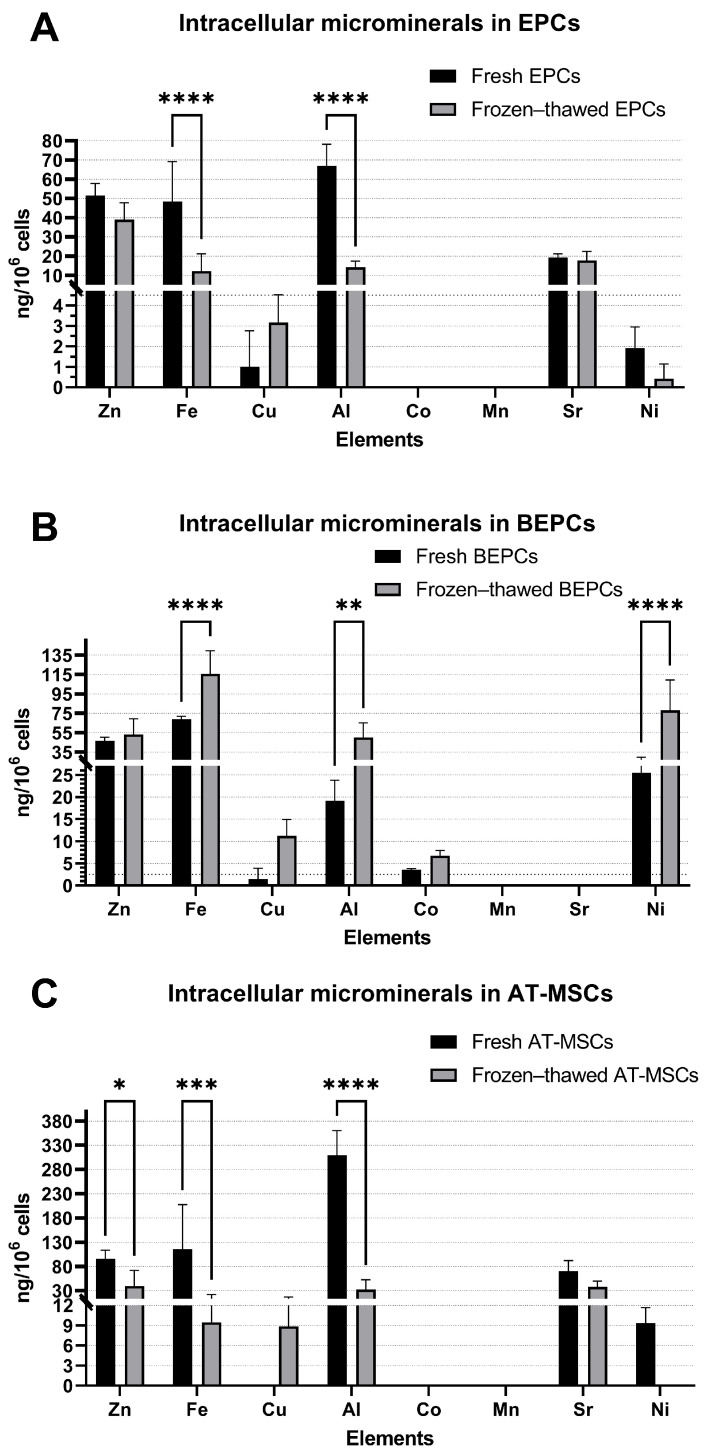
Effect of cryopreservation on the composition of microminerals in the rabbit primary cell lines (**A**–**C**). EPCs—rabbit endothelial progenitor cells derived from peripheral blood, BEPCs—rabbit endothelial progenitor cells derived from bone marrow, AT-MSCs—rabbit adipose-tissue derived mesenchymal stem cells. The data from three independent cell cultures for each cell line are expressed as the means ± SD; *—the difference is statistically significant at *p* < 0.05; **—the difference is statistically significant at *p* < 0.01; ***—the difference is statistically significant at *p* < 0.001; ****—the difference is statistically significant at *p* < 0.0001. Measurements for Co (fresh and frozen–thawed EPCs (**A**) and AT-MSCs (**C**)), Mn (fresh and frozen–thawed EPCs (**A**), BEPCs (**B**) and AT-MSCs (**C**)), Cu (fresh AT-MSCs (**C**)), and Ni (frozen–thawed AT-MSCs (**C**)) were under detection limits.

The composition of several microminerals was notably affected by the cryopreservation process in all three rabbit cell lines. A decrease (*p* < 0.0001) in the level of Fe and Al was noticed in frozen–thawed EPCs in comparison to fresh samples (Figure 3A), whereas levels of other microminerals (Zn, Cu, Sr, and Ni) did not change. The concentration of Co and Mn were under the detection limit of the instrument. On the contrary, levels of Fe, Al, and Ni increased (*p* < 0.01) in frozen–thawed BEPCs compared to fresh samples (Figure 3B). The concentration of other microminerals was not affected by cryopreservation of BEPCs, although their levels also increased (Zn, Cu, and Co), but insignificantly. The levels of Mn and Sr have not been detected in these samples. In cryopreserved AT-MSCs, the concentration of some microminerals (Zn, Fe, and Al) decreased (*p* < 0.05). Unfortunately, Cu has not been detected in fresh samples, while on the other hand, Ni has been observed only in fresh samples. In addition, levels of Co and Mn have not been observed in either fresh or frozen–thawed samples (Figure 3C).

## 4. Discussion

In general, cells should be preserved for later applications using hypothermic conditions for short-term or cryopreserved for long-term storage. However, extended cold storage may cause injury to cells as a negative consequence of oxidative stress, mechanical damage due to the formation of ice crystals, changed physical attributes of cell structures, changes in osmosis or ion homeostasis, etc. Current assays for the quality control of cells exposed to cold storage include viability methods evaluating plasma membrane integrity, proliferation or differentiation assay, etc. However, a lack of studies analyzed the changes in elemental concentrations or electrolyte composition of cells during their cold storage [30,31,32].

In this study, the ICP-OES method was applied to analyze the elemental composition of three different rabbit primary cell lines and the possible change triggered by long-term- storage. The ICP-OES technique has already been used to determine the cellular influx or efflux of specific elements in different experimental studies [15,16,17,18,19,20,21] or to define the elemental profile of human adrenal tissue and blood samples [14]. However, as far as we know, this is the first study to specify the content of elements in rabbit primary cells and their fluctuations after the cryopreservation process. Here, we analyzed the content of macrominerals (Ca, Na, K, and Mg) and microminerals (Zn, Fe, Cu, Al, Co, Mn, Sr, and Ni) in rabbit EPCs, BEPCs, and AT-MSCs. We found some significant differences in the concentration of macro and microminerals among the freshly cultured rabbit stem/progenitor cell lines (Figure 1), with AT-MSCs showing the highest concentrations of several measured elements. This might indicate that the elemental composition of specific cell lines depends on the biological source or tissue type from which they originate or even on the composition of used culture media. For example, significant differences were also found in the concentration of macroelements (Ca, Na, K, and Mg) and microelements (Mn, Co, Cu, Zn, As, Se, Cd, Pb, and U) of healthy adrenal tissue in comparison to adenomatous adrenal tissue [14]. On the other hand, significant changes in the elemental concentrations of Na, Cl, K, or Ca were observed in pancreas tissue samples preserved in several different culture media at 4 °C for 4 h compared to control freshly isolated tissue [32]. Moreover, the content of the elements assessed in this study by X-ray microanalysis also fluctuated in different time intervals (after 4, 8, and 12 h) of short-term cold storage. According to these findings, the authors of the study concluded that the composition of the culture or storage medium is the main factor that determines the concentration of ions in cells after cold storage. However, they compared the stored samples with control freshly isolated tissue and not with tissue cultured for a certain time. On the contrary, X-ray microanalysis revealed changes in intracellular elements such as increased Na and Cl and decreased K in human hematopoietic cells (U937 cell line) preserved in different storage mediums and for different time intervals (1, 2, 4, 8, and 24 h of short-term storage) in comparison to cultured cells [30,31]. Similarly, the long-term cold storage (cryopreservation in liquid nitrogen) of rabbit cells in our study significantly decreased the concentration of K in rabbit EPCs and BEPCs as well as Ca in AT-MSCs. On the other hand, the content of Na increased significantly only in frozen–thawed BEPCs (Figure 2).

It seems that cryopreservation may also affect the content of intracellular microminerals since a significant decrease was noticed in the level of Fe and Al for frozen–thawed EPCs, and AT-MSCs and Zn levels were also significantly reduced in AT-MSCs (Figure 3). Contrarily, the content of Fe and Al together with Ni significantly increased after long-term storage in BEPCs. Unfortunately, the lack of studies focused on the elemental composition of cells under culture or storage conditions does not allow us to compare our findings with the experiments published elsewhere. Nevertheless, micronutrients and microminerals themselves are very important components of cell cultures. The deficiency or excess of Cu, Fe, Mg, Se, or Zn may result in genomic instability and impaired viability of cultured cells [33]. For example, increased concentration of Cu may induce cytotoxicity, formation of ROS, reduction of mitochondrial activity and overall viability of cells, as well as damage to DNA [34,35]. Higher or lower content of Fe may inhibit the synthesis of DNA during cell proliferation or induce genotoxicity [36,37]. Decreased Mg concentrations lead to the inhibition of cell growth and proliferation and can accelerate the senescence of cells [38,39,40]. Abnormal levels of Se cause cell death and apoptosis [41,42]. A high or even low content of Zn may trigger oxidative damage to DNA and inhibit the growth and viability of cells [43,44,45]. High levels of aluminum may increase the incidence of double-strand breaks in DNA, aneuploidy, or abnormal cell cycle [46]. It has been observed that low Sr doses stimulate the formation of bones, whereas high amounts of Sr trigger defects in bone mineralization [8,47]. Increased Ni exposure induces carcinogenesis and allergy mainly because of oxidative stress and mitochondrial dysfunctions in cells [9,48].

Despite the changes in macro and micromineral composition in cryopreserved cells, we can assume that further re-culture of frozen–thawed rabbit primary cells in a specific culture medium may restore the intracellular mineral balance. This hypothesis is supported by our previous studies, in which frozen–thawed rabbit MSCs or EPCs successfully proliferate or differentiate after long-term storage in liquid nitrogen [28,49]. Anyway, further information is required to decide whether the changes in concentration of elements after cryopreservation may negatively affect their further applications.

## 5. Conclusions

Here, we used ICP-OES analysis to determine the concentration of macro- and microminerals in three rabbit primary cell lines before and after cryopreservation. The contents of several analyzed elements were significantly changed, probably due to the long-term cold storage of rabbit cells. Finally, we can conclude that the ICP-OES technique might be helpful for the analysis of the basic elemental composition of animal cells in any condition, fresh or frozen–thawed. Nevertheless, additional studies are needed to reveal the possible impact of cryopreservation on cell fate by changing the content of intracellular minerals.

## Figures and Tables

**Table 1:** Operating instrument conditions used for ICP-OES analysis of minerals contained in cells.

Analysis Parameters	Value
Plasma RF Power	1150 W
Purge Gas Flow	Normal
Auxiliary Gas Flow	0.50 L/min
Coolant Gas Flow	12 L/min
Nebulizer Gas Flow	0.45 L/min
Nebulizer Gas Pressure	120 kPa
Pump Speed	50 rpm

## Data Availability

The data presented in this study are available in the article.

## References

[1] Saha S.K., Pathak N.N. (2021). Chemicals of Life and Chemical Reactions in the Animal Cells. Fundamentals of Animal Nutrition.

[2] Nieder R., Benbi D.K., Reichl F.X. (2018). Microelements and Their Role in Human Health. Soil Components and Human Health.

[3] Spears J. (1999). Reevaluation of the metabolic essentiality of the minerals—Review. Asian-Australas. J. Anim. Sci..

[4] Combs G.F. (2012). Geological impacts on nutrition. Essentials of Medical Geology: Revised Edition.

[5] Fraga C.G. (2005). Relevance, essentiality and toxicity of trace elements in human health. Mol. Asp. Med..

[6] Exley C. (2003). A biogeochemical cycle for aluminium?. J. Inorg. Biochem..

[7] Exley C., Kretsinger R.H., Uversky V.N., Permyakov E.A. (2013). Aluminum in Biological Systems. Encyclopedia of Metalloproteins.

[8] Pilmane M., Salma-Ancane K., Loca D., Locs J., Berzina-Cimdina L. (2017). Strontium and strontium ranelate: Historical review of some of their functions. Mater. Sci. Eng. C-Mater. Biol. Appl..

[9] Zambelli B., Ciurli S., Sigel A., Sigel H., Sigel R.K.O. (2013). Nickel and Human Health. Interrelations Between Essential Metal Ions and Human Diseases.

[10] Denkhaus E., Salnikow K. (2002). Nickel essentiality, toxicity, and carcinogenicity. Crit. Rev. Oncol./Hematol..

[11] Bulska E., Wagner B. (2016). Quantitative aspects of inductively coupled plasma mass spectrometry. Philos. Trans. R. Soc. A-Math. Phys. Eng. Sci..

[12] Douvris C., Vaughan T., Bussan D., Bartzas G., Thomas R. (2023). How ICP-OES changed the face of trace element analysis: Review of the global application landscape. Sci. Total Environ..

[13] Boss C.B., Fredeen K.J. (2004). Concepts, Instrumentation and Techniques in Inductively Coupled Plasma Optical Emission Spectrometry.

[14] Jagodic J., Rovcanin B., Krstic D., Paunovic I., Zivaljevic V., Manojlovic D., Stojsavljevic A. (2021). Elemental profiling of adrenal adenomas in solid tissue and blood samples by ICP-MS and ICP-OES. Microchem. J..

[15] Egger A., Rappel C., Jakupec M., Hartinger C., Heffeter P., Keppler B. (2009). Development of an experimental protocol for uptake studies of metal compounds in adherent tumor cells. J. Anal. At. Spectrom..

[16] Gligorijevic N., Arandelovic S., Filipovic L., Jakovljevic K., Jankovic R., Grguric-Sipka S., Ivanovic I., Radulovic S., Tesic Z. (2012). Picolinate ruthenium(II)-arene complex with in vitro antiproliferative and antimetastatic properties: Comparison to a series of ruthenium(II)-arene complexes with similar structure. J. Inorg. Biochem..

[17] Thompson E., Graham E., MacNeill C., Young M., Donati G., Wailes E., Jones B., Levi-Polyachenko N. (2014). Differential response of MCF7, MDA-MB-231, and MCF 10A cells to hyperthermia, silver nanoparticles and silver nanoparticle-induced photothermal therapy. Int. J. Hyperth..

[18] Andreas K., Georgieva R., Ladwig M., Mueller S., Notter M., Sittinger M., Ringe J. (2012). Highly efficient magnetic stem cell labeling with citrate-coated superparamagnetic iron oxide nanoparticles for MRI tracking. Biomaterials.

[19] Nedopil A., Klenk C., Kim C., Liu S., Wendland M., Golovko D., Schuster T., Sennino B., McDonald D., Daldrup-Link H. (2010). MR Signal Characteristics of Viable and Apoptotic Human Mesenchymal Stem Cells in Matrix-Associated Stem Cell Implants for Treatment of Osteoarthritis. Investig. Radiol..

[20] Zheng B., von See M., Yu E., Gunel B., Lu K., Vazin T., Schaffer D., Goodwill P., Conolly S. (2016). Quantitative Magnetic Particle Imaging Monitors the Transplantation, Biodistribution, and Clearance of Stem Cells In Vivo. Theranostics.

[21] Gong Z., Zhou J., Li H., Gao Y., Xu C., Zhao S., Chen Y., Cai W., Wu J. (2015). Curcumin suppresses NLRP3 inflammasome activation and protects against LPS-induced septic shock. Mol. Nutr. Food Res..

[22] Marquez-Curtis L., Janowska-Wieczorek A., McGann L., Elliott J. (2015). Mesenchymal stromal cells derived from various tissues: Biological, clinical and cryopreservation aspects. Cryobiology.

[23] Sieme H., Oldenhof H., Wolkers W. (2016). Mode of action of cryoprotectants for sperm preservation. Anim. Reprod. Sci..

[24] Meseguer M., Garrido N., Martínez-Conejero J., Simón C., Pellicer A., Remohí J. (2004). Mitochondrial activity in the susceptibility for cryodamage after a cycle of freezing and thawing Role of cholesterol, calcium, and thawing. Fertil. Steril..

[25] Shon Y.H. (1993). Analysis of the Proteoglycan Content in Fresh and Cryopreserved Porcine Cardiovascular Tissues.

[26] Villalobos-Olivera A., Martínez J., Escalante D., Martínez-Montero M.E., Sershen N., Lorenzo J.C. (2021). Cryopreservation of pineapple shoot tips does not affect mineral contents of regenerated plants. Acta Physiol. Plant..

[27] Kovac M., Vasicek J., Kulikova B., Bauer M., Curlej J., Balazi A., Chrenek P. (2017). Different RNA and protein expression of surface markers in rabbit amniotic fluid-derived mesenchymal stem cells. Biotechnol. Prog..

[28] Vasicek J., Balazi A., Bauer M., Svoradova A., Tirpakova M., Tomka M., Chrenek P. (2021). Molecular Profiling and Gene Banking of Rabbit EPCs Derived from Two Biological Sources. Genes.

[29] Tirpakova M., Vasicek J., Svoradova A., Balazi A., Tomka M., Bauer M., Makarevich A., Chrenek P. (2021). Phenotypical Characterization and Neurogenic Differentiation of Rabbit Adipose Tissue-Derived Mesenchymal Stem Cells. Genes.

[30] Arrebola F., Cañizares F.J., Cubero M.A., Serrano M.M., Robles M.A., Fernández-Segura E. (2008). Ultrastructural and Intracellular Elemental Composition Analysis of Human Hematopoietic Cells During Cold Storage in Preservation Solutions.

[31] Fernández-Segura E., Arrebola F., Cubero M.A., Cañizares F.J., Robles M.A., Navarrete P. (2008). Changes in Intracellular Sodium, Chlorine, and Potassium Content in Hematopoietic Cells After Hypotermic Storage.

[32] Kozlova I., Roomans G. (2003). Preservation of pancreas tissue during cold storage assessed by X-ray microanalysis. Am. J. Transplant..

[33] Arigony A., de Oliveira I., Machado M., Bordin D., Bergter L., Pra D., Henriques J. (2013). The Influence of Micronutrients in Cell Culture: A Reflection on Viability and Genomic Stability. BioMed Res. Int..

[34] Seth R., Yang S., Cho S., Sabean M., Roberts E. (2004). In vitro assessment of copper-induced toxicity in the human hepatoma line, Hep G. Toxicol. In Vitro.

[35] Grillo C., Reigosa M., de Mele M. (2010). Does over-exposure to copper ions released from metallic copper induce cytotoxic and genotoxic effects on mammalian cells?. Contraception.

[36] Lima P., Vasconcellos M., Montenegro R., Sombra C., Bahia M., Costa-Lotufo L., Pessoa C., Moraes M., Burbano R. (2008). Genotoxic and cytotoxic effects of iron sulfate in cultured human lymphocytes treated in different phases of cell cycle. Toxicol. In Vitro.

[37] Knoebel Y., Weise A., Glei M., Sendt W., Claussen U., Pool-Zobel B. (2007). Ferric iron is genotoxic in non-transformed and preneoplastic human colon cells. Food Chem. Toxicol..

[38] Maier J., Malpuech-Brugère C., Zimowska W., Rayssiguier Y., Mazur A. (2004). Low magnesium promotes endothelial cell dysfunction:: Implications for atherosclerosis, inflammation and thrombosis. Biochim. Biophys. Acta-Mol. Basis Dis..

[39] Sgambato A., Wolf F., Faraglia B., Cittadini A. (1999). Magnesium depletion causes growth inhibition, reduced expression of cyclin D1, and increased expression of p27^Kip1^ in normal but not in transformed mammary epithelial cells. J. Cell. Physiol..

[40] Killilea D., Ames B. (2008). Magnesium deficiency accelerates cellular senescence in cultured human fibroblasts. Proc. Natl. Acad. Sci. USA.

[41] Hoefig C., Renko K., Köhrle J., Birringer M., Schomburg L. (2011). Comparison of different selenocompounds with respect to nutritional value vs. toxicity using liver cells in culture. J. Nutr. Biochem..

[42] Zeng H., Wu M., Botnen J. (2009). Methylselenol, a Selenium Metabolite, Induces Cell Cycle Arrest in G1 Phase and Apoptosis via the Extracellular-Regulated Kinase 1/2 Pathway and Other Cancer Signaling Genes. J. Nutr..

[43] Ho E., Ames B. (2002). Low intracellular zinc induces oxidative DNA damage, disrupts p53, NFκB, and AP1 DNA binding, and affects DNA repair in a rat glioma cell line. Proc. Natl. Acad. Sci. USA.

[44] Sharif R., Thomas P., Zalewski P., Graham R., Fenech M. (2011). The effect of zinc sulphate and zinc carnosine on genome stability and cytotoxicity in the WIL2-NS human lymphoblastoid cell line. Mutat. Res.-Genet. Toxicol. Environ. Mutagen..

[45] Sharif R., Thomas P., Zalewski P., Fenech M. (2012). Zinc deficiency or excess within the physiological range increases genome instability and cytotoxicity, respectively, in human oral keratinocyte cells. Genes Nutr..

[46] Tenan M., Nicolle A., Moralli D., Verbouwe E., Jankowska J., Durin M., Green C., Mandriota S., Sappino A. (2021). Aluminum Enters Mammalian Cells and Destabilizes Chromosome Structure and Number. Int. J. Mol. Sci..

[47] Grynpas M., Hamilton E., Cheung R., Tsouderos Y., Deloffre P., Hott M., Marie P. (1996). Strontium increases vertebral bone volume in rats at a low dose that does not induce detectable mineralization defect. Bone.

[48] Genchi G., Carocci A., Lauria G., Sinicropi M., Catalano A. (2020). Nickel: Human Health and Environmental Toxicology. Int. J. Environ. Res. Public Health.

[49] Vasicek J., Kovac M., Balazi A., Kulikova B., Tomkova M., Olexikova L., Curlej J., Bauer M., Schnabl S., Hilgarth M. (2020). Combined approach for characterization and quality assessment of rabbit bone marrow-derived mesenchymal stem cells intended for gene banking. New Biotechnol..

